# An Upwind Interior Penalty DG Scheme for Solute Transport in 2D Variable-Order Mobile–Immobile Model

**DOI:** 10.3390/e28090997

**Published:** 2026-09-06

**Authors:** Leilei Wei, Lijie Liu, Xindong Zhang

**Affiliations:** 1 School of Mathematics and Information Science, North Minzu University, Yinchuan 750021, China; 2 College of Big Data Statistics, Guizhou University of Finance and Economics, Guiyang 550025, China

**Keywords:** solute transport, variable-order fractional derivative, mobile–immobile model, discontinuous Galerkin method, two-dimensional problem, error estimate

## Abstract

This paper develops and rigorously analyzes a fully discrete upwind interior penalty discontinuous Galerkin (IPDG) scheme for simulating solute transport in two-dimensional variable-order fractional mobile–immobile media. The temporal variable-order Caputo derivative is discretized via a Grünwald–Letnikov approximation in conjunction with a first-order backward difference, while the spatial discretization employs an IPDG method featuring an upwind numerical flux for the convection term and a penalty formulation for the diffusion operator. Under the physically relevant assumption of a divergence-free velocity field, we establish the unconditional stability of the proposed scheme. A comprehensive error analysis in the $L^{2}$ norm yields a convergence rate of $O(\Delta t+h^{\min(k+1,s)-\chi -1/2})$, explicitly linking the polynomial degree *k*, solution regularity *s*, and the penalty variant χ. Numerical experiments in two dimensions are conducted to verify the accuracy and robustness of the proposed scheme in simulating anomalous transport phenomena in subsurface environments.

## 1. Introduction

Unlike integer-order classical derivatives, fractional nonlocal operators are capable of representing long-range memory and spatial inhomogeneity present in complicated systems. Hence, they provide a more precise description of anomalous diffusion, propagation dynamics, and numerous physical phenomena in porous substances, viscoelastic materials, and biological tissues relative to conventional models [1,2]. Over recent years, fractional calculus has gained widespread interest in many disciplines, such as hydrology, physics, finance, and engineering.

Owing to the nonlocal character and history dependence inherent in fractional operators, exact (analytical) solutions of fractional differential equations are often challenging or infeasible to derive. Thus, substantial research efforts have been directed toward constructing efficient and trustworthy numerical algorithms for such equations. Representative approaches comprise finite difference schemes [3,4,5,6], finite element techniques [7,8,9], discontinuous Galerkin methods [10,11,12,13], meshless strategies [14,15], and spectral schemes [16,17,18], all of which serve as powerful instruments for studying fractional partial differential equations (FPDEs). For a broader perspective, the reader may consult [19,20,21] and the citations therein.

A wide range of dynamical systems exhibit fractional characteristics that can vary with time or location, rendering variable-order calculus a feasible modeling framework. In contrast to constant-order fractional derivatives—which presume a fixed memory exponent—variable-order operators permit the order to change in a dynamic manner, thereby capturing evolving physical or statistical features. Such derivatives find application in mathematical modeling across various areas, e.g., anomalous diffusion in heterogeneous porous media, viscoelasticity featuring time-dependent rheological parameters, biological signal processing, and financial systems subject to regime transitions. Coimbra [22] originally proposed a physically meaningful variable-order derivative for viscoelastic problems, whereas Lorenzo and Hartley [23] together with Ramirez and Coimbra [24] subsequently conducted systematic analyses of distinct definitions and their characteristics. The adaptability of variable-order models facilitates more faithful representations of complex systems whose governing dynamics change over time or space, for instance, solute transport in fractured media where the anomalous diffusion degree may depend on the distance from the injection source or the evolution of the fracture network.

A variable-order time-fractional mobile–immobile advection–dispersion model describes solute transport within porous and fractured media. In the classical mobile–immobile framework, the total concentration is decomposed into a mobile phase and an immobile phase, and the two phases exchange mass through a first-order rate process. After eliminating the immobile-phase concentration, the mobile-phase equation contains a convolution memory term that accounts for the delayed release of solute from the immobile zone. In the fractional generalization, this memory kernel is replaced by a power-law kernel, which yields the fractional derivative $D_{t}^{\alpha (t)}C$ in the governing equation. The variable order α(t) reflects the temporal evolution of the mass-transfer rate or the heterogeneity of the immobile zone. Detailed physical justifications and field applications of this modeling framework can be found in [25,26,27]. For solving the mobile–immobile advection–dispersion system, Zhang et al. [28] put forward an implicit Euler approximation in 2013 and verified its unconditional stability. In 2017, employing reproducing kernel theory along with a collocation technique, Jiang et al. [29] developed a numerical solver for the same advection–dispersion model. In that same year, Liu et al. [30] devised and examined a second-order finite difference scheme to handle quasilinear fractal mobile–immobile transport equations using novel fractional derivatives. Using radial basis function approximations, Golbabai et al. [31] solved a time-fractional mobile–immobile advection–dispersion problem on a bounded domain, deriving an approximate solution via meshless techniques built upon radial basis functions. For the two-dimensional case of the time variable-order fractional mobile–immobile advection–dispersion model, Saffarian and Mohebbi [32] designed an efficient numerical approach. They constructed a fully discrete formulation employing a second-order temporal discretization together with the Legendre spectral element method in space, and they established that the scheme remains unconditionally stable. A spectral collocation strategy using sixth-kind Chebyshev polynomials was put forward by Sadri et al. [33] to treat numerical solutions of variable-order time-fractional mobile–immobile advection equations.

In the present work, we examine a two-dimensional time-dependent variable-order fractional mobile–immobile advection–dispersion problem of the following form:

(1) $$\begin{array}{cc} \frac{\partial C(x,y,t)}{\partial t}+D_{t}^{\alpha (t)}C\left(x,y,t\right)+V\cdot \nabla C\left(x,y,t\right) & =\Delta C(x,y,t)+f(x,y,t),\;(x,y,t)\in \Omega \times (0,T],\\ C(x,y,t) & =0,\;\mathrm{on}\;\partial \Omega ,\\ C(x,y,0) & =0,\;\mathrm{in}\;\Omega . \end{array}$$

with $0<\underline{\alpha }\leq \alpha \left(t\right)\leq \bar{\alpha }\leq 1$. The prescribed convection field is denoted by $V=(V_{1},V_{2})$. In what follows, we suppose ***V*** is divergence-free in $\bar{\Omega }$, so that

$$\nabla \cdot V=0\;\mathrm{in}\;\bar{\Omega },$$

and assume the additional regularity $V\in L^{\infty }\;\left(\left[0,T\right];\;W^{1,\infty }{\left(\bar{\Omega }\right)}^{2}\right).$

The variable-order Caputo fractional derivative appearing in Equation (1) is given by [8]

(2) $$D_{t}^{\alpha (t)}C\left(x,y,t\right)=\frac{1}{\Gamma (1-\alpha (t))}\int_{0}^{t}\frac{\partial C(x,y,z)}{\partial z}\frac{dz}{{\left(t-z\right)}^{\alpha (t)}},\;t>0.$$

The governing Equation (1) can be derived from a classical two-phase mobile–immobile transport model. Let $C_{m}$ and $C_{im}$ denote the mobile and immobile concentrations, and let $\theta_{m},\theta_{im}$ be the corresponding volumetric fractions. The mass balance and first-order exchange equations are

$$\theta_{m}\frac{\partial C_{m}}{\partial t}+\theta_{im}\frac{\partial C_{im}}{\partial t}=\nabla \cdot \left(D\nabla C_{m}\right)-V\cdot \nabla C_{m},\;\frac{\partial C_{im}}{\partial t}=\lambda \left(C_{m}-C_{im}\right),$$

where λ is the mass-transfer coefficient and *D* is the dispersion tensor. Solving the second equation with $C_{im}\left(0\right)=0$ gives

$$C_{im}\left(t\right)=\lambda \int_{0}^{t}e^{-\lambda (t-s)}C_{m}\left(s\right)\;ds,$$

and substituting this into the first equation yields an integro-differential equation for $C_{m}$ with a convolution memory term. In the fractional generalization, the exponential kernel is replaced by the power-law kernel

$$\frac{t^{-\alpha (t)}}{\Gamma (1-\alpha (t))},$$

so that the memory term becomes proportional to the variable-order Caputo derivative $D_{t}^{\alpha (t)}C_{m}$. After normalizing parameters and assuming isotropic diffusion D=I, one obtains Equation (1) with $C_{m}$ relabeled as *C*. This derivation shows that the fractional derivative represents delayed release from the immobile zone, and the variable order α(t) models temporal changes in the mass-transfer process.

Section 2 presents the notation, projection operators, and theoretical results needed for the subsequent analysis. The construction of a numerical scheme for Problem (1) using the IPDG technique is described in Section 3, where we also establish unconditional stability and convergence of the proposed method. Section 4 presents several numerical experiments to illustrate the performance and robustness of the proposed scheme. Finally, Section 5 concludes with some remarks.

## 2. Notation and Auxiliary Results

Assume that $\Omega \subset R^{2}$ is a polygonal domain, and let $T_{h}$ denote a conforming triangulation of Ω consisting of non-overlapping triangular cells *K*. Specifically,

$$T_{h}=\left\{K\subset \Omega :K\;\mathrm{is}\;\mathrm{triangular},\;\bar{\Omega }=\underset{K\in T_{h}}{⋃}\bar{K}\right\}.$$

We define the global mesh parameter by

$$h=\max_{K\in T_{h}}h_{K},$$

with $h_{K}$ the diameter of *K*, and we denote by $E_{h}$ the collection of all edges of $T_{h}$, which splits into $E_{h}=E_{h}^{\mathrm{int}}\cup E_{h}^{\mathrm{ext}}$; here, $E_{h}^{\mathrm{int}}$ and $E_{h}^{\mathrm{ext}}$ are the interior and boundary edge sets, respectively.

Assign to each edge $e\in E_{h}$ a unit normal vector $n_{e}$. For an interior edge $e\in E_{h}^{\mathrm{int}}$, let $K^{-}$ and $K^{+}$ be the two adjacent elements, and choose the orientation so that $n_{e}$ points from $K^{-}$ to $K^{+}$; this choice is arbitrary but remains fixed for each edge. For a scalar function $v\in H^{1}\left(T_{h}\right)$, the average and jump across an interior edge $e\in E_{h}^{\mathrm{int}}$ are defined by

$$\left\{v\right\}:=\frac{1}{2}\left(v^{-}+v^{+}\right),\;\left[v\right]:=v^{-}-v^{+},$$

where $v^{\pm }$ denote the traces of *v* from $K^{\pm }$ on *e*. On boundary edges $e\in E_{h}^{\mathrm{ext}}$, we take $n_{e}$ to be the outward unit normal to ∂Ω, and we set

$${\left\{v\right\}:=v|}_{K}{,\;\left[v\right]:=v|}_{K},$$

where *K* is the unique element adjacent to *e*. Due to the homogeneous Dirichlet boundary condition, the exterior trace on boundary edges is set to zero, i.e., ${v|}_{\mathrm{ext}}=0$. Consequently, the jump on a boundary edge reduces to ${\left[v\right]=v|}_{K}$.

Take a fixed integer k∈N. Let $P_{k}\left(K\right)$ denote the space of polynomials on each element $K\in T_{h}$ whose total degree does not exceed *k*. We denote by $V_{k}\left(T_{h}\right)$ the discontinuous Galerkin finite element space consisting of piecewise polynomials of degree at most *k*, i.e.,

$$V_{k}\left(T_{h}\right):=\left\{v_{h}\in L^{2}\left(\Omega \right):v_{h}{|}_{K}\in P_{k}\left(K\right),\;\forall K\in T_{h}\right\}.$$

**Lemma** **1** ([34])**.** *Under the condition 0<α(t)<1, the left-sided Caputo fractional derivative defined by*

Dtα(t)Cw(t)=1Γ(1−α(t))∫0tw′(s)(t−s)α(t)ds

*is identical to the Riemann–Liouville fractional derivative*

Dtα(t)RLw(t)=1Γ(1−α(t))ddt∫0tw(s)(t−s)α(t)ds

*whenever w(0)=0.*

**Lemma** **2** ([34] Grünwald formula)**.** *Define*

$$Q^{s+b}\left(R\right)=\left\{w\in L^{1}\left(R\right):\int_{-\infty }^{\infty }{\left(1+\left|\psi \right|\right)}^{s+b}\;\left|\hat{w}\left(\psi \right)\right|\;d\psi <+\infty \right\},$$

*where s,b∈R with s+b>0, and s+b denotes the sum of the two real parameters s and b, which appears as the exponent in the weight ${\left(1+|\psi |\right)}^{s+b}$.*

*If $w\in Q^{1+b}\left(R\right)$, then the following holds:*

$$\frac{1}{\Gamma (1-\alpha (t))}\frac{d}{dt}\int_{-\infty }^{t}\frac{w(s)}{{\left(t-s\right)}^{\alpha (t)}}ds=\frac{1}{{\left(\Delta t\right)}^{\alpha (t)}}\sum_{j=0}^{\infty }\rho_{j}^{\alpha (t)}w\left(t-\left(j-\lambda \right)\Delta t\right)+O\left(\Delta t\right),$$

*where λ is an integer and $\rho_{j}^{\alpha (t)}={\left(-1\right)}^{j}\left(\begin{array}{c} \alpha (t)\\ j \end{array}\right).$*

**Lemma** **3.** Let $u\in C^{2}\left[0,T\right]$ with u(0)=0, and let $\alpha \in C^{1}\left[0,T\right]$ satisfy $0<\alpha_{*}\leq \alpha \left(t\right)\leq \alpha^{*}<1$ for all t∈[0,T]. Define the truncation error

(3) $$R^{n}:=D_{t}^{\alpha (t_{n})}u\left(t_{n}\right)-\frac{1}{{\left(\Delta t\right)}^{\alpha (t_{n})}}\sum_{k=0}^{n}\rho_{k}^{\alpha (t_{n})}u\left(t_{n-k}\right),$$

where $\rho_{k}^{\alpha (t_{n})}$ are the Grünwald coefficients. Then, there exists a constant C>0, depending on u, $\alpha_{*}$, $\alpha^{*}$, and $∥\alpha^{\prime }{∥}_{L^{\infty }}$, but independent of Δt, such that

$$|R^{n}|\leq C\;\Delta t.$$

**Proof.**  Since u(0)=0, Lemma 1 implies that the Caputo derivative equals the Riemann–Liouville derivative:

Dtα(tn)u(tn)=Dtα(tn)RLu(tn)=1Γ(1−α(tn))ddt∫0tnu(s)(tn−s)α(tn)ds.

Extend *u* by zero to (−∞,0). For the extended function, the shifted Grünwald approximation of the Riemann–Liouville derivative reads

Dtα(tn)RLu(tn)=1(Δt)α(tn)∑k=0∞ρkα(tn)u(tn−k)+En,

where, under the stated regularity assumptions, the error term satisfies

$$|E^{n}|\leq C_{1}\Delta t$$

with $C_{1}$ independent of Δt (see, e.g., [34,35]). Because u≡0 on (−∞,0), the infinite series truncates to

$$\sum_{k=0}^{n}\rho_{k}^{\alpha (t_{n})}u\left(t_{n-k}\right),$$

since all terms with k>n correspond to negative time arguments and vanish. Therefore,

$$R^{n}=E^{n},$$

and hence $|R^{n}|\leq C\Delta t$ with $C=C_{1}$. This completes the proof. □

**Lemma** **4** ([35])**.** *For 0<α(t)<1, the coefficients $\rho_{j}^{\alpha (t)}$ possess the following properties:*

$$\rho_{0}^{\alpha (t)}=1,\;\rho_{1}^{\alpha (t)}=-\alpha \left(t\right)\leq 0,$$

$$\rho_{2}^{\alpha (t)}\leq \rho_{3}^{\alpha (t)}\leq \rho_{4}^{\alpha (t)}\leq \cdots \leq 0,$$

$$\sum_{k=0}^{\infty }\rho_{k}^{\alpha (t)}=0,\;\sum_{k=0}^{n}\rho_{k}^{\alpha (t)}\geq 0\;\;\left(n\geq 1\right),$$

*and they can be computed recursively via*

$$\rho_{0}^{\alpha (t)}=1,\;\rho_{k}^{\alpha (t)}=\left(1-\frac{\alpha (t)+1}{k}\right)\rho_{k-1}^{\alpha (t)}\;\;\left(k\geq 1\right).$$

**Lemma** **5** ([35])**.** *There exists a positive constant ς>0 such that*

$$\frac{1}{{\left(\Delta t\right)}^{\alpha (t)}}\sum_{k=0}^{n-1}\rho_{k}^{\alpha (t)}\geq \varsigma >0.$$

Throughout this work, we employ a generic positive constant C whose value may vary from one occurrence to another.

## 3. The Scheme

For the discretization in time, the interval [0,T] is split uniformly into M∈N subintervals with step size Δt=T/M. Denote the discrete time nodes by $t_{n}=n\Delta t$ for n=0,1,…,M. The terms $C_{t}$ and $D_{t}^{\alpha (t_{n})}C$ are approximated at $t_{n}$ in the following manner:

(4) $$\begin{array}{cc} \frac{\partial C(x,y,t)}{\partial t} & =\frac{C\left(x,y,t_{n}\right)-C\left(x,y,t_{n-1}\right)}{\Delta t}+\gamma_{1}^{n}\left(x\right), \end{array}$$

Meanwhile, to approximate the variable-order Caputo fractional derivative, we employ the Grünwald–Letnikov formulation. Invoking Lemmas 1 and 2 yields

(5) $$\begin{array}{cc} D_{t}^{\alpha (t_{n})}C\left(x,y,t_{n}\right) & =\frac{1}{\Gamma (1-\alpha \left(t_{n}\right))}\int_{0}^{t_{n}}\frac{\partial C(x,y,s)}{\partial s}\frac{1}{{\left(t_{n}-s\right)}^{\alpha (t_{n})}}ds\\ \; & =\frac{1}{{\left(\Delta t\right)}^{\alpha (t_{n})}}\sum_{k=0}^{n}\rho_{k}^{\alpha (t_{n})}C\left(x,y,(n-k)\Delta t\right)+\gamma_{2}^{n}\left(x\right), \end{array}$$

where $\gamma^{n}\left(x\right)=\gamma_{1}^{n}\left(x\right)+\gamma_{2}^{n}\left(x\right)$ denotes the temporal truncation error. From Lemma 3 and the standard first-order backward difference approximation, there exists a constant C>0, independent of Δt and *h*, such that

$$∥\gamma^{n}\left(x\right)∥\leq C\Delta t.$$

Let $C_{h}^{n}\in V_{h}^{k}$ denote the discrete approximation of $C(\cdot ,t_{n})$, and set $f^{n}\left(x\right)=f\left(x,y,t_{n}\right)$. The fully discrete formulation reads: find $C_{h}^{n}\in V_{h}^{k}$ such that for every test function $v\in V_{h}^{k}$,

(6) $$\begin{array}{cc} \frac{1}{\Delta t}\int_{\Omega }C_{h}^{n}v\;dx & +\frac{\rho_{0}^{\alpha (t_{n})}}{{\left(\Delta t\right)}^{\alpha (t_{n})}}\int_{\Omega }C_{h}^{n}v\;dx-\int_{T_{h}}VC_{h}^{n}\cdot \nabla v\;dx+\int_{E_{h}}V\cdot n_{e}\hat{C_{h}^{n}}\left[v\right]\;ds\\ \; & +\int_{T_{h}}\nabla C_{h}^{n}\cdot \nabla v\;dx-\int_{E_{h}}\left\{\nabla C_{h}^{n}\cdot n_{e}\right\}\left[v\right]\;ds+\xi \int_{E_{h}}\left\{\nabla v\cdot n_{e}\right\}\left[C_{h}^{n}\right]\;ds\\ \; & +\int_{E_{h}}\frac{\delta }{{\left|e\right|}^{\kappa }}\left[C_{h}^{n}\right]\left[v\right]\;ds\\ = & \frac{1}{\Delta t}\int_{\Omega }C_{h}^{n-1}v\;dx+\frac{1}{{\left(\Delta t\right)}^{\alpha (t_{n})}}\sum_{k=1}^{n}\left(-\rho_{k}^{\alpha (t_{n})}\right)\int_{\Omega }C_{h}^{n-k}v\;dx+\int_{\Omega }f^{n}v\;dx, \end{array}$$

The parameter ξ∈{−1,0,1} [36] determines the type of interior penalty method:

$$\left\{\begin{array}{cc} \xi =-1: & \mathrm{symmetric}\;\mathrm{interior}\;\mathrm{penalty}\;\mathrm{Galerkin}\;\left(\mathrm{SIPG}\right)\;\mathrm{method},\\ \xi =0: & \mathrm{incomplete}\;\mathrm{interior}\;\mathrm{penalty}\;\mathrm{Galerkin}\;\left(\mathrm{IIPG}\right)\;\mathrm{method},\\ \xi =1: & \mathrm{non}\text{-}\mathrm{symmetric}\;\mathrm{interior}\;\mathrm{penalty}\;\mathrm{Galerkin}\;\left(\mathrm{NIPG}\right)\;\mathrm{method}. \end{array}\right.$$

The numerical flux $\hat{C_{h}^{n}}$, which arises from integration by parts along element boundaries, is required to be single-valued on each interface and must be chosen appropriately to guarantee stability. The upwind flux is chosen as follows:

(7) $$C_{h}^{n,\mathrm{upwind}}=\left\{\begin{array}{cc} C_{h}^{n}{|}_{K_{1}}, & \mathrm{if}\;V\cdot n_{e}\geq 0,\\ C_{h}^{n}{|}_{K_{2}}, & \mathrm{if}\;V\cdot n_{e}<0. \end{array}\right.$$

For a boundary edge $e\in E_{h}^{\mathrm{ext}}$, the exterior trace is $C_{h}^{n}{|}_{\mathrm{ext}}=0$; hence, the upwind flux is defined as

$$\hat{C_{h}^{n}}=\left\{\begin{array}{cc} C_{h}^{n}{|}_{K}, & V\cdot n_{e}\geq 0,\\ 0, & V\cdot n_{e}<0, \end{array}\right.$$

where *K* is the unique element adjacent to *e*.

We now introduce the following bilinear forms:

$$\begin{array}{cc} \Phi_{\xi }\left(p,q\right)= & \int_{T_{h}}\nabla p\cdot \nabla q\;dx-\int_{E_{h}}\left\{\nabla p\cdot n_{e}\right\}\left[q\right]\;ds+\xi \int_{E_{h}}\left[\nabla q\cdot n_{e}\right]\left\{p\right\}\;ds+\int_{E_{h}}\frac{\delta }{{\left|e\right|}^{\kappa }}\left[p\right]\left[q\right]\;ds,\\ \Lambda \left(p,q\right)= & -\int_{T_{h}}Vp\cdot \nabla q\;dx+\int_{E_{h}}V\cdot n_{e}\hat{p}\left[q\right]\;ds. \end{array}$$

**Lemma** **6** ([36])**.** *Define the energy norm by*

$${∥\phi ∥}_{H}^{2}:=\int_{T_{h}}{\left|\nabla \phi \right|}^{2}\;dx+\int_{E_{h}}\frac{\delta }{{\left|e\right|}^{\kappa }}{\left[\phi \right]}^{2}\;ds.$$

*For the non-symmetric interior penalty Galerkin (NIPG) method, i.e., ξ=1 in (6), the bilinear form $\Phi_{\xi }$ is coercive: there exists a positive constant $m_{1}>0$, independent of h, such that*

$$\Phi_{\xi }\left(\phi ,\phi \right)\geq m_{1}{∥\phi ∥}_{H}^{2}\;\forall \phi \in V_{k}\left(T_{h}\right).$$

*For the incomplete IPG (ξ=0) and symmetric IPG (ξ=−1) methods, coercivity is guaranteed provided the penalty parameter δ is chosen sufficiently large.*

### 3.1. Stability Analysis

In the stability analysis, we assume, without loss of generality, that the source term vanishes, i.e., f=0.

**Theorem** **1.** *The fully discrete DG scheme (6) is unconditionally stable, and $C_{h}^{n}$ satisfies*

(8) $$\begin{array}{cc} \; & ∥C_{h}^{n}∥\leq ∥C_{h}^{0}∥,\;n=1,2,\ldots ,M. \end{array}$$

**Proof.**  Choosing $v=C_{h}^{n}$ in (6) yields

(9) $$\begin{array}{cc} \frac{1}{\Delta t}{∥C_{h}^{n}∥}^{2} & +\frac{\rho_{0}^{\alpha (t_{n})}}{{\left(\Delta t\right)}^{\alpha (t_{n})}}{∥C_{h}^{n}∥}^{2}+\Lambda \left(C_{h}^{n},C_{h}^{n}\right)+\Phi_{\xi }\left(C_{h}^{n},C_{h}^{n}\right)\\ = & \frac{1}{\Delta t}\int_{\Omega }C_{h}^{n-1}C_{h}^{n}\;dx+\frac{1}{{\left(\Delta t\right)}^{\alpha (t_{n})}}\sum_{k=1}^{n}\left(-\rho_{k}^{\alpha (t_{n})}\right)\int_{\Omega }C_{h}^{n-k}C_{h}^{n}\;dx. \end{array}$$

Now, examine the term $\Lambda (C_{h}^{n},C_{h}^{n})$. By virtue of the upwind flux (7) together with the divergence-free property ∇·V=0, we obtain

(10) $$\begin{array}{cc} \Lambda (C_{h}^{n},C_{h}^{n}) & =-\int_{T_{h}}VC_{h}^{n}\cdot \nabla C_{h}^{n}\;dx+\int_{E_{h}}V\cdot n_{e}\;C_{h}^{n,\mathrm{upwind}}\left[C_{h}^{n}\right]\;ds\\ & =\sum_{e\in E_{h}}\int_{e}V\cdot n_{e}\left(C_{h}^{n,\mathrm{upwind}}\left[C_{h}^{n}\right]-\frac{1}{2}{\left[C_{h}^{n}\right]}^{2}\right)ds\\ & =\sum_{e\in E_{h}}\int_{e}V\cdot n_{e}\left(C_{h}^{n,\mathrm{upwind}}\left[C_{h}^{n}\right]-\left\{C_{h}^{n}\right\}\left[C_{h}^{n}\right]\right)ds\\ & =\frac{1}{2}\sum_{e\in E_{h}}\int_{e}\left|V\cdot n_{e}\right|{\left[C_{h}^{n}\right]}^{2}\;ds\geq 0. \end{array}$$

In deriving (10), we split the edge set into interior and boundary edges. For boundary edges $e\in E_{h}^{\mathrm{ext}}$, the exterior trace is zero, so the jump satisfies $\left[C_{h}^{n}\right]=C_{h}^{n}{|}_{K}$. Using the boundary upwind flux defined above, a direct computation verifies that the identity

$$\int_{e}V\cdot n_{e}\left(C_{h}^{n,\mathrm{upwind}}\left[C_{h}^{n}\right]-\frac{1}{2}{\left[C_{h}^{n}\right]}^{2}\right)ds=\frac{1}{2}\int_{e}\left|V\cdot n_{e}\right|{\left[C_{h}^{n}\right]}^{2}\;ds$$

holds for each boundary edge as well. Hence, the final equality in (10) is valid over the entire edge set $E_{h}$.

Invoking Lemma 6 yields the coercivity estimate

(11) $$\Phi_{\xi }\left(C_{h}^{n},C_{h}^{n}\right)\geq m_{1}{∥C_{h}^{n}∥}_{H}^{2},$$

with some constant $m_{1}>0$.

From Lemma 4, we obtain

(12) $$-\rho_{n}^{\alpha (t_{n})}\leq \sum_{k=0}^{n-1}\rho_{k}^{\alpha (t_{n})}.$$

Applying the Cauchy–Schwarz inequality transforms (9) into

(13) $$\begin{array}{cc} & \frac{1}{\Delta t}{∥C_{h}^{n}∥}^{2}+\frac{1}{{\left(\Delta t\right)}^{\alpha (t_{n})}}\rho_{0}^{\alpha (t_{n})}{∥C_{h}^{n}∥}^{2}+m_{1}{∥C_{h}^{n}∥}_{H}^{2}\\ & \leq \frac{1}{\Delta t}∥C_{h}^{n-1}∥∥C_{h}^{n}∥+\frac{1}{{\left(\Delta t\right)}^{\alpha (t_{n})}}\sum_{k=1}^{n-1}\left(-\rho_{k}^{\alpha (t_{n})}\right)∥C_{h}^{n-k}∥∥C_{h}^{n}∥\\ & +\frac{1}{{\left(\Delta t\right)}^{\alpha (t_{n})}}\left(-\rho_{n}^{\alpha (t_{n})}\right)∥C_{h}^{0}∥∥C_{h}^{n}∥\\ & \leq \frac{1}{\Delta t}∥C_{h}^{n-1}∥∥C_{h}^{n}∥+\frac{1}{{\left(\Delta t\right)}^{\alpha (t_{n})}}\sum_{k=1}^{n-1}\left(-\rho_{k}^{\alpha (t_{n})}\right)∥C_{h}^{n-k}∥∥C_{h}^{n}∥\\ & +\frac{1}{{\left(\Delta t\right)}^{\alpha (t_{n})}}\sum_{k=0}^{n-1}\rho_{k}^{\alpha (t_{n})}∥C_{h}^{0}∥∥C_{h}^{n}∥. \end{array}$$

Dividing both sides by $∥C_{h}^{n}∥$ (which is non-zero; otherwise, the inequality holds trivially) gives

(14) $$\begin{array}{cc} & \left(\frac{1}{\Delta t}+\frac{1}{{\left(\Delta t\right)}^{\alpha (t_{n})}}\rho_{0}^{\alpha (t_{n})}\right)∥C_{h}^{n}∥\\ & \leq \frac{1}{\Delta t}∥C_{h}^{n-1}∥+\frac{1}{{\left(\Delta t\right)}^{\alpha (t_{n})}}\sum_{k=1}^{n-1}\left(-\rho_{k}^{\alpha (t_{n})}\right)∥C_{h}^{n-k}∥+\frac{1}{{\left(\Delta t\right)}^{\alpha (t_{n})}}\sum_{k=0}^{n-1}\rho_{k}^{\alpha (t_{n})}∥C_{h}^{0}∥. \end{array}$$

We proceed by induction on *n*. For n=1, Equation (14) gives

(15) $$∥C_{h}^{1}∥\leq ∥C_{h}^{0}∥.$$

Assume that the following inequalities hold:

(16) $$\begin{array}{c} ∥C_{h}^{m}∥\leq ∥C_{h}^{0}∥,\;m=1,2,3,\ldots ,n-1. \end{array}$$

Our goal is to show $∥C_{h}^{n}∥\leq ∥C_{h}^{0}∥$. From (14), we deduce

(17) $$\begin{array}{cc} & \left(\frac{1}{\Delta t}+\frac{1}{{\left(\Delta t\right)}^{\alpha (t_{n})}}\rho_{0}^{\alpha (t_{n})}\right)∥C_{h}^{n}∥\\ & \leq \left(\frac{1}{\Delta t}+\frac{1}{{\left(\Delta t\right)}^{\alpha (t_{n})}}\sum_{k=1}^{n-1}\left(-\rho_{k}^{\alpha (t_{n})}\right)+\frac{1}{{\left(\Delta t\right)}^{\alpha (t_{n})}}\sum_{k=0}^{n-1}\rho_{k}^{\alpha (t_{n})}\right)∥C_{h}^{0}∥\\ & =\left(\frac{1}{\Delta t}+\frac{1}{{\left(\Delta t\right)}^{\alpha (t_{n})}}\rho_{0}^{\alpha (t_{n})}\right)∥C_{h}^{0}∥, \end{array}$$

and therefore

$$\begin{array}{c} ∥C_{h}^{n}∥\leq ∥C_{h}^{0}∥. \end{array}$$

□

### 3.2. Error Estimate

We now turn to error estimates measured in the $L^{2}$ norm. To this end, we introduce a projection operator P acting on the exact solution ω [36]:

(18) $$\forall \omega \in H^{s}\left(T_{h}\right),\;\Phi_{\xi }\left(\omega -P\omega ,v\right)=0.$$

**Lemma** **7** ([36])**.** *Suppose ω lies in $L^{2}\left(0,T;H^{s}\left(T_{h}\right)\right)$ with s>3/2. Then, the following bound holds:*

(19) $${∥\omega -P\omega ∥}_{H}\leq Ch^{\min(k+1,s)-1}{∥\omega \left(t\right)∥}_{H^{s}\left(T_{h}\right)}.$$

*Moreover, when* Ω *is convex, the* $L^{2}$ *norm error estimates satisfy*

(20) $${∥\omega -P\omega ∥}_{L^{2}\left(\Omega \right)}\leq Ch^{\min(k+1,s)-\chi }{∥\omega \left(t\right)∥}_{H^{s}\left(T_{h}\right)},$$

*with χ=0 for SIPG and χ=1 for NIPG and IIPG.*

**Theorem** **2.** *Suppose C(x,t) denotes the exact solution to problem (1), and let $C_{h}^{n}$ denote the numerical approximation obtained via the DG scheme (6). Then, there holds*

$$∥C\left(x,t_{n}\right)-C_{h}^{n}∥\leq C(\Delta t+h^{\min\left(k+1,s\right)-\chi -\frac{1}{2}}),$$

*where C is a positive constant independent of both h and Δt.*

**Proof.**  Let

(21) $$e_{c}^{n}=C\left(x,y,t_{n}\right)-C_{h}^{n}=\xi_{c}^{n}-\eta_{c}^{n},\;\xi_{c}^{n}=Pe_{c}^{n},\;\eta_{c}^{n}=PC\left(x,y,t_{n}\right)-C\left(x,y,t_{n}\right),$$

where $\eta_{c}^{n}$ is bounded using Lemma 7. In what follows, we concentrate on estimating $\xi_{c}^{n}$.

The following error equation can be derived:

(22) $$\begin{array}{cc} \frac{1}{\Delta t}\int_{\Omega }e_{c}^{n}v\;dx & +\frac{1}{{\left(\Delta t\right)}^{\alpha (t_{n})}}\rho_{0}^{\alpha (t_{n})}\int_{\Omega }e_{c}^{n}v\;dx+\Lambda \left(e_{c}^{n},v\right)+\Phi_{\xi }\left(e_{c}^{n},v\right)+\int_{\Omega }\gamma^{n}\left(x\right)v\;dx\\ & =\frac{1}{\Delta t}\int_{\Omega }e_{c}^{n-1}v\;dx+\frac{1}{{\left(\Delta t\right)}^{\alpha (t_{n})}}\sum_{k=1}^{n}\left(-\rho_{k}^{\alpha (t_{n})}\right)\int_{\Omega }e_{c}^{n-k}v\;dx. \end{array}$$

Choosing the test function $v=\xi_{c}^{n}$ and substituting (21) into (22) yields

(23) $$\begin{array}{cc} \frac{1}{\Delta t}{∥\xi_{c}^{n}∥}^{2} & +\frac{1}{{\left(\Delta t\right)}^{\alpha (t_{n})}}\rho_{0}^{\alpha (t_{n})}{∥\xi_{c}^{n}∥}^{2}+\Lambda \left(\xi_{c}^{n},\xi_{c}^{n}\right)+\Phi_{\xi }\left(\xi_{c}^{n},\xi_{c}^{n}\right)\\ & =\frac{1}{\Delta t}\int_{\Omega }\eta_{c}^{n}\xi_{c}^{n}\;dx+\frac{1}{{\left(\Delta t\right)}^{\alpha (t_{n})}}\rho_{0}^{\alpha (t_{n})}\int_{\Omega }\eta_{c}^{n}\xi_{c}^{n}\;dx\\ & +\frac{1}{\Delta t}\int_{\Omega }\xi_{c}^{n-1}\xi_{c}^{n}\;dx-\frac{1}{\Delta t}\int_{\Omega }\eta_{c}^{n-1}\xi_{c}^{n}\;dx-\int_{\Omega }\gamma^{n}\left(x\right)\xi_{c}^{n}\;dx\\ & +\frac{1}{{\left(\Delta t\right)}^{\alpha (t_{n})}}\sum_{k=1}^{n}\left(-\rho_{k}^{\alpha (t_{n})}\right)\int_{\Omega }\xi_{c}^{n-k}\xi_{c}^{n}\;dx\\ & -\frac{1}{{\left(\Delta t\right)}^{\alpha (t_{n})}}\sum_{k=1}^{n}\left(-\rho_{k}^{\alpha (t_{n})}\right)\int_{\Omega }\eta_{c}^{n-k}\xi_{c}^{n}\;dx+\Lambda \left(\eta_{c}^{n},\xi_{c}^{n}\right). \end{array}$$

Invoking Lemma 6 provides the following lower bound for $\Phi_{\xi }$:

(24) $$\Phi_{\xi }\left(\xi_{c}^{n},\xi_{c}^{n}\right)\geq m_{1}{∥\xi_{c}^{n}∥}_{H}^{2}.$$

Furthermore, we have

(25) $$\Lambda \left(\xi_{c}^{n},\xi_{c}^{n}\right)\geq 0.$$

To handle edge contributions, we apply the trace inequality,

$${∥v∥}_{L^{2}\left(e\right)}^{2}\leq C\left(h_{K}^{-1}{∥v∥}_{L^{2}\left(K\right)}^{2}+h_{K}{∥\nabla v∥}_{L^{2}\left(K\right)}^{2}\right).$$

Together with the approximation properties of P, this implies ${∥{\hat{\eta }}_{c}^{n}∥}_{L^{2}\left(e\right)}\leq Ch^{\min\left(k+1,s\right)-\chi -\frac{1}{2}}$, which is used to bound $\Lambda (\eta_{c}^{n},\xi_{c}^{n})$ as follows:

(26) $$\begin{array}{cc} \Lambda (\eta_{c}^{n},\xi_{c}^{n}) & \leq |\left(V\eta_{c}^{n},\nabla \xi_{c}^{n}\right)|+\left|\sum_{e\in E_{h}}\int_{e}V\cdot n_{e}\hat{\eta_{c}^{n}}\left[\xi_{c}^{n}\right]\;ds\right|\\ & \leq {∥V∥}_{\infty }\sum_{e\in E_{h}}{∥\hat{\eta_{c}^{n}}∥}_{L^{2}\left(e\right)}{∥\left[\xi_{c}^{n}\right]∥}_{L^{2}\left(e\right)}\\ & \leq {\epsilon ∥V∥}_{\infty }^{2}\sum_{e\in E_{h}}{∥\hat{\eta_{c}^{n}}∥}_{L^{2}\left(e\right)}^{2}+\frac{1}{4\epsilon }\sum_{e\in E_{h}}{∥\left[\xi_{c}^{n}\right]∥}_{L^{2}\left(e\right)}^{2}\\ & \leq {\epsilon ∥V∥}_{\infty }^{2}h^{2\min(k+1,s)-2\chi -1}+\frac{1}{4\epsilon }{∥\xi_{c}^{n}∥}_{L^{2}\left(E_{h}\right)}^{2}. \end{array}$$

Using (12) together with the Cauchy–Schwarz inequality, we obtain the following estimate:

(27) $$\begin{array}{cc} \frac{1}{\Delta t}{∥\xi_{c}^{n}∥}^{2} & +\frac{1}{{\left(\Delta t\right)}^{\alpha (t_{n})}}\rho_{0}^{\alpha (t_{n})}{∥\xi_{c}^{n}∥}^{2}+m_{1}{∥\xi_{c}^{n}∥}_{H}^{2}\\ & \leq \frac{1}{\Delta t}\left(∥\eta_{c}^{n}∥+∥\eta_{c}^{n-1}∥\right)∥\xi_{c}^{n}∥+\frac{1}{\Delta t}∥\xi_{c}^{n-1}∥∥\xi_{c}^{n}∥+∥\gamma^{n}\left(x\right)∥∥\xi_{c}^{n}∥\\ & +\frac{1}{{\left(\Delta t\right)}^{\alpha (t_{n})}}\sum_{k=1}^{n-1}\left(-\rho_{k}^{\alpha (t_{n})}\right)∥\xi_{c}^{n-k}∥∥\xi_{c}^{n}∥+\frac{1}{{\left(\Delta t\right)}^{\alpha (t_{n})}}\sum_{k=0}^{n-1}\rho_{k}^{\alpha (t_{n})}∥\xi_{c}^{0}∥∥\xi_{c}^{n}∥\\ & +\frac{1}{{\left(\Delta t\right)}^{\alpha (t_{n})}}\sum_{k=1}^{n-1}\left(-\rho_{k}^{\alpha (t_{n})}\right)∥\eta_{c}^{n-k}∥∥\xi_{c}^{n}∥+\frac{1}{{\left(\Delta t\right)}^{\alpha (t_{n})}}\sum_{k=0}^{n-1}\rho_{k}^{\alpha (t_{n})}∥\eta_{c}^{0}∥∥\xi_{c}^{n}∥\\ & +\frac{1}{{\left(\Delta t\right)}^{\alpha (t_{n})}}\rho_{0}^{\alpha (t_{n})}∥\eta_{c}^{n}∥∥\xi_{c}^{n}∥+{\epsilon ∥V∥}_{\infty }^{2}h^{2\min(k+1,s)-2\chi -1}+\frac{1}{4\epsilon }{∥\xi_{c}^{n}∥}_{L^{2}\left(E_{h}\right)}^{2}. \end{array}$$

Consequently,

(28) $$\begin{array}{cc} & \frac{1}{\Delta t}{∥\xi_{c}^{n}∥}^{2}+\frac{1}{{\left(\Delta t\right)}^{\alpha (t_{n})}}\rho_{0}^{\alpha (t_{n})}{∥\xi_{c}^{n}∥}^{2}+m_{1}{∥\xi_{c}^{n}∥}_{H}^{2}\\ & \leq (\frac{1}{\Delta t}\left(∥\eta_{c}^{n}∥+∥\eta_{c}^{n-1}∥\right)+\frac{1}{\Delta t}∥\xi_{c}^{n-1}∥+∥\gamma^{n}\left(x\right)∥\\ & +\left(\frac{1}{{\left(\Delta t\right)}^{\alpha (t_{n})}}\sum_{k=1}^{n-1}\left(-\rho_{k}^{\alpha (t_{n})}\right)\right)\left(∥\xi_{c}^{n-k}∥+∥\eta_{c}^{n-k}∥\right)\\ & +\left(\frac{1}{{\left(\Delta t\right)}^{\alpha (t_{n})}}\sum_{k=0}^{n-1}\rho_{k}^{\alpha (t_{n})}\right)\left(∥\xi_{c}^{0}∥+∥\eta_{c}^{0}∥\right)+\frac{1}{{\left(\Delta t\right)}^{\alpha (t_{n})}}\rho_{0}^{\alpha (t_{n})}∥\eta_{c}^{n}∥)∥\xi_{c}^{n}∥\\ & +{\epsilon ∥V∥}_{\infty }^{2}h^{2\min(k+1,s)-2\chi -1}+\frac{1}{4\epsilon }{∥\xi_{c}^{n}∥}_{L^{2}\left(E_{h}\right)}^{2}. \end{array}$$

There exists a positive constant ζ>0 such that

$$-\left(\rho_{1}^{\alpha (t_{n})}+\rho_{2}^{\alpha (t_{n})}+\cdots +\rho_{n-1}^{\alpha (t_{n})}\right)\leq \rho_{0}^{\alpha (t_{n})}\leq -\zeta \left(\rho_{1}^{\alpha (t_{n})}+\rho_{2}^{\alpha (t_{n})}+\cdots +\rho_{n-1}^{\alpha (t_{n})}\right).$$

Applying Lemma 5 and setting $\theta =\frac{1}{\Delta t}+\frac{1}{{\left(\Delta t\right)}^{\alpha (t_{n})}}\rho_{0}^{\alpha (t_{n})}$, inequality (28) transforms into

(29) $$\begin{array}{cc} \frac{\theta }{2}{∥\xi_{c}^{n}∥}^{2} & \leq \frac{1}{2\theta }(\frac{1}{\Delta t}\left(∥\eta_{c}^{n}∥+∥\eta_{c}^{n-1}∥\right)+\frac{1}{\Delta t}∥\xi_{c}^{n-1}∥\\ & +\left(\frac{1}{{\left(\Delta t\right)}^{\alpha (t_{n})}}\sum_{k=1}^{n-1}\left(-\rho_{k}^{\alpha (t_{n})}\right)\right)\left(∥\xi_{c}^{n-k}∥+∥\eta_{c}^{n-k}∥+∥\eta_{c}^{n}∥\right)\\ & +\left(\frac{1}{{\left(\Delta t\right)}^{\alpha (t_{n})}}\sum_{k=0}^{n-1}\rho_{k}^{\alpha (t_{n})}\right)\left(∥\xi_{c}^{0}∥+∥\eta_{c}^{0}∥+\frac{1}{\varsigma }∥\gamma^{n}\left(x\right)∥\right){)}^{2}\\ & +\epsilon h^{2\min(k+1,s)-2\chi -1}+\frac{1}{4\epsilon }{∥\xi_{c}^{n}∥}_{L^{2}\left(E_{h}\right)}^{2}, \end{array}$$

using the elementary relation

$$a^{2}+b^{2}\leq {\left(a+b\right)}^{2},\;ab\geq 0,$$

we arrive at the following bound:

(30) $$\begin{array}{cc} \theta ∥\xi_{c}^{n}∥ & \leq \frac{1}{\Delta t}\left(∥\eta_{c}^{n}∥+∥\eta_{c}^{n-1}∥\right)+\frac{1}{\Delta t}∥\xi_{c}^{n-1}∥\\ & +\left(\frac{1}{{\left(\Delta t\right)}^{\alpha (t_{n})}}\sum_{k=1}^{n-1}\left(-\rho_{k}^{\alpha (t_{n})}\right)\right)\left(∥\xi_{c}^{n-k}∥+∥\eta_{c}^{n-k}∥+∥\eta_{c}^{n}∥+\frac{{\epsilon ∥V∥}_{\infty }}{\zeta }Ch^{\min\left(k+1,s\right)-\chi -\frac{1}{2}}\right)\\ & +\left(\frac{1}{{\left(\Delta t\right)}^{\alpha (t_{n})}}\sum_{k=0}^{n-1}\rho_{k}^{\alpha (t_{n})}\right)\left(∥\xi_{c}^{0}∥+∥\eta_{c}^{0}∥+\frac{1}{\varsigma }∥\gamma^{n}\left(x\right)∥\right). \end{array}$$

For the case n=1, Equation (30) reduces to

(31) $$\begin{array}{cc} \left(\frac{1}{\Delta t}+\frac{1}{{\left(\Delta t\right)}^{\alpha (t_{1})}}\rho_{0}^{\alpha (t_{1})}\right)∥\xi_{c}^{1}∥ & \leq \frac{1}{\Delta t}\left(∥\eta_{c}^{1}∥+∥\eta_{c}^{0}∥\right)+\frac{1}{\Delta t}∥\xi_{c}^{0}∥\\ & +\left(\frac{1}{{\left(\Delta t\right)}^{\alpha (t_{1})}}\rho_{0}^{\alpha (t_{1})}\right)\left(∥\xi_{c}^{0}∥+∥\eta_{c}^{0}∥+\frac{1}{\varsigma }∥\gamma^{1}\left(x\right)∥\right). \end{array}$$

Noting that $∥\xi_{c}^{0}∥=0$ and invoking (20), we obtain

(32) $$\begin{array}{cc} ∥\xi_{c}^{1}∥ & \leq C\left(\Delta t+h^{\min\left(k+1,s\right)-\chi -\frac{1}{2}}\right). \end{array}$$

We now assume that the following estimate holds:

(33) $$\begin{array}{c} ∥\xi_{c}^{m}∥\leq C\left(\Delta t+h^{\min\left(k+1,s\right)-\chi -\frac{1}{2}}\right),\;m=1,2,\ldots ,n-1. \end{array}$$

Observe that

$$\frac{1}{{\left(\Delta t\right)}^{\alpha (t_{n})}}\sum_{k=0}^{n-1}\rho_{k}^{\alpha (t_{n})}+\frac{1}{{\left(\Delta t\right)}^{\alpha (t_{n})}}\sum_{k=1}^{n-1}\left(-\rho_{k}^{\alpha (t_{n})}\right)=\frac{1}{{\left(\Delta t\right)}^{\alpha (t_{n})}}\rho_{0}^{\alpha (t_{n})}.$$

Combining (30) with the induction hypothesis (33) yields

(34) $$\begin{array}{cc} & \left(\frac{1}{\Delta t}+\frac{1}{{\left(\Delta t\right)}^{\alpha (t_{n})}}\rho_{0}^{\alpha (t_{n})}\right)∥\xi_{c}^{n}∥\leq \frac{1}{\Delta t}C\left(\Delta t+h^{\min\left(k+1,s\right)-\chi -\frac{1}{2}}\right)\\ & +\frac{1}{{\left(\Delta t\right)}^{\alpha (t_{n})}}\sum_{k=1}^{n-1}\left(-\rho_{k}^{\alpha (t_{n})}\right)C\left(\Delta t+h^{\min\left(k+1,s\right)-\chi -\frac{1}{2}}\right)\\ & +\frac{1}{{\left(\Delta t\right)}^{\alpha (t_{n})}}\sum_{k=0}^{n-1}\rho_{k}^{\alpha (t_{n})}C\left(\Delta t+h^{\min\left(k+1,s\right)-\chi -\frac{1}{2}}\right), \end{array}$$

hence,

$$∥\xi_{c}^{n}∥\leq C\left(\Delta t+h^{\min\left(k+1,s\right)-\chi -\frac{1}{2}}\right).$$

Finally, applying the triangle inequality together with the approximation properties of the projection completes the proof of Theorem 2. □

**Remark** **1.** *The spatial error bound in Theorem 2 is conservative for smooth solutions. The $-\frac{1}{2}$ loss in the exponent arises from the use of trace inequalities when estimating the convective edge terms involving $\eta_{c}^{n}=PC-C$. Specifically, terms of the form $\sum_{e\in E_{h}}{∥{\hat{\eta }}_{c}^{n}∥}_{L^{2}\left(e\right)}$ are controlled by the projection error on element boundaries, which scales like $h^{\min\left(k+1,s\right)-\chi -\frac{1}{2}}$ because the trace inequality introduces an additional factor $h^{-1/2}$ relative to the $L^{2}\left(\Omega \right)$ bound. This is a technical consequence of the analysis rather than an inherent property of the scheme.*

*For sufficiently smooth exact solutions and for the symmetric interior penalty variant, a sharper treatment of the boundary terms is possible if one exploits higher regularity of the projection error on edges or uses local $L^{\infty }$ estimates. Under such stronger assumptions, the suboptimal half-order loss can be removed, potentially recovering the optimal rate $O(h^{k+1})$. However, establishing this refined estimate rigorously is beyond the scope of the present paper. Therefore, Theorem 2 states the conservative bound that holds under the general regularity assumptions considered here, while the numerical experiments in Section 4 exhibit the sharper $O(h^{k+1})$ behavior for smooth manufactured solutions.*

## 4. Numerical Experiment

The main purpose of this section is to verify the theoretical convergence orders established in Section 3 under controlled conditions. For this reason, we employ smooth manufactured solutions on a square domain. Although the proposed scheme is designed to accommodate triangular meshes, local polynomial enrichment, and advection-dominated transport through the upwind flux, the present numerical tests are not intended to simulate realistic field-scale transport involving heterogeneous media, sharp fronts, or strongly advection-dominated conditions. Such applications will be investigated in future work. In the mesh generation process, the parameter SE specifies the number of subdivisions along each boundary edge, whereas *h* denotes the maximum edge length of the resulting triangular elements.

Consider the problem (1) on the domain Ω=[0,1]×[0,1], and the source term is

$$\begin{array}{cc} f(x,y,t)= & \left[3t^{2}+\frac{6t^{3-\alpha (t)}}{\Gamma (4-\alpha (t))}+8\pi^{2}t^{3}\right]\sin\left(2\pi x\right)\sin\left(2\pi y\right)\\ \; & +2\pi t^{3}\left[V_{1}\cos\left(2\pi x\right)\sin\left(2\pi y\right)+V_{2}\sin\left(2\pi x\right)\cos\left(2\pi y\right)\right]. \end{array}$$

with the exact solution given by

$$C_{\mathrm{exact}}\left(x,y,t\right)=t^{3}\sin\left(2\pi x\right)\sin\left(2\pi y\right).$$

To evaluate the performance of the numerical scheme, we examine various spatial and temporal discretization parameters. The approximation errors are quantified using both the $L^{\infty }$ and $L^{2}$ norms.

For a given error Error(h) corresponding to mesh size *h*, the convergence rate order is computed as:

$$order=\frac{\log\left(\frac{Error(h_{1})}{Error(h_{2})}\right)}{\log\left(\frac{h_{1}}{h_{2}}\right)}.$$

Piecewise $P^{1}$ basis functions are adopted for spatial approximation. We take κ=1, δ=10000. Table 1 and Table 2 report the numerical errors and associated convergence rates for two distinct velocity fields, V=(1,1) and V=(−x+0.5,y−0.5), respectively, under different α(t) specifications. In Table 1, with V=(1,1), the $L^{2}$ and $L^{\infty }$ errors diminish monotonically as *h* decreases. The obtained convergence orders remain near two for both α(t)=0.8+0.1sint and $\alpha \left(t\right)=1-0.5e^{-t}$, confirming second-order spatial accuracy. Table 2 presents analogous results for V=(−x+0.5,y−0.5), where the scheme again yields convergence rates approximately equal to two across all tested α(t).

**Figure 1:**
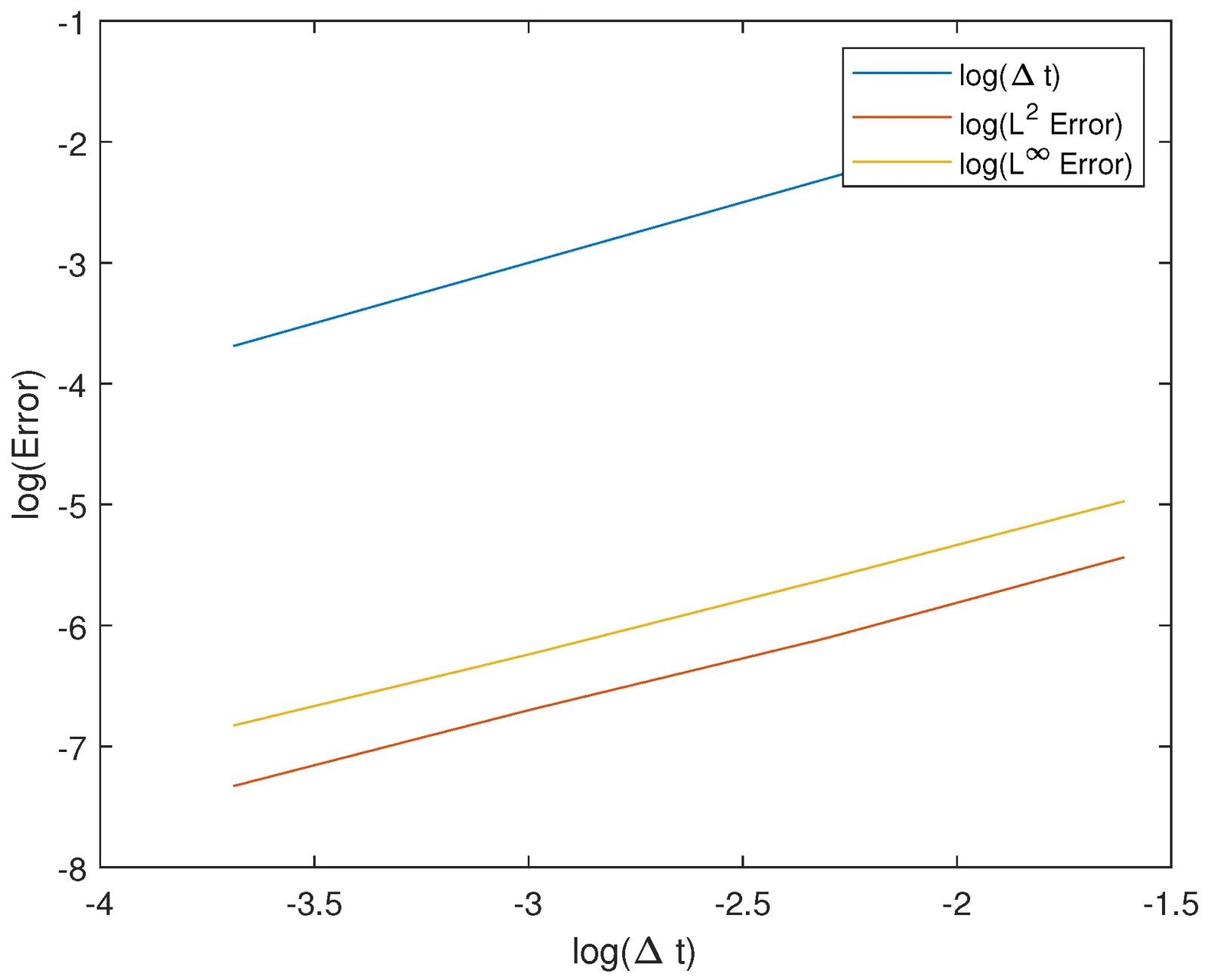
Error in $L^{2}$ and $L^{\infty }$ norm versus Δt, order for $\alpha \left(t\right)=\frac{{\left(\sin t\right)}^{2}+2}{5},\;SE=40,\;k=1$.

**Figure 2:**
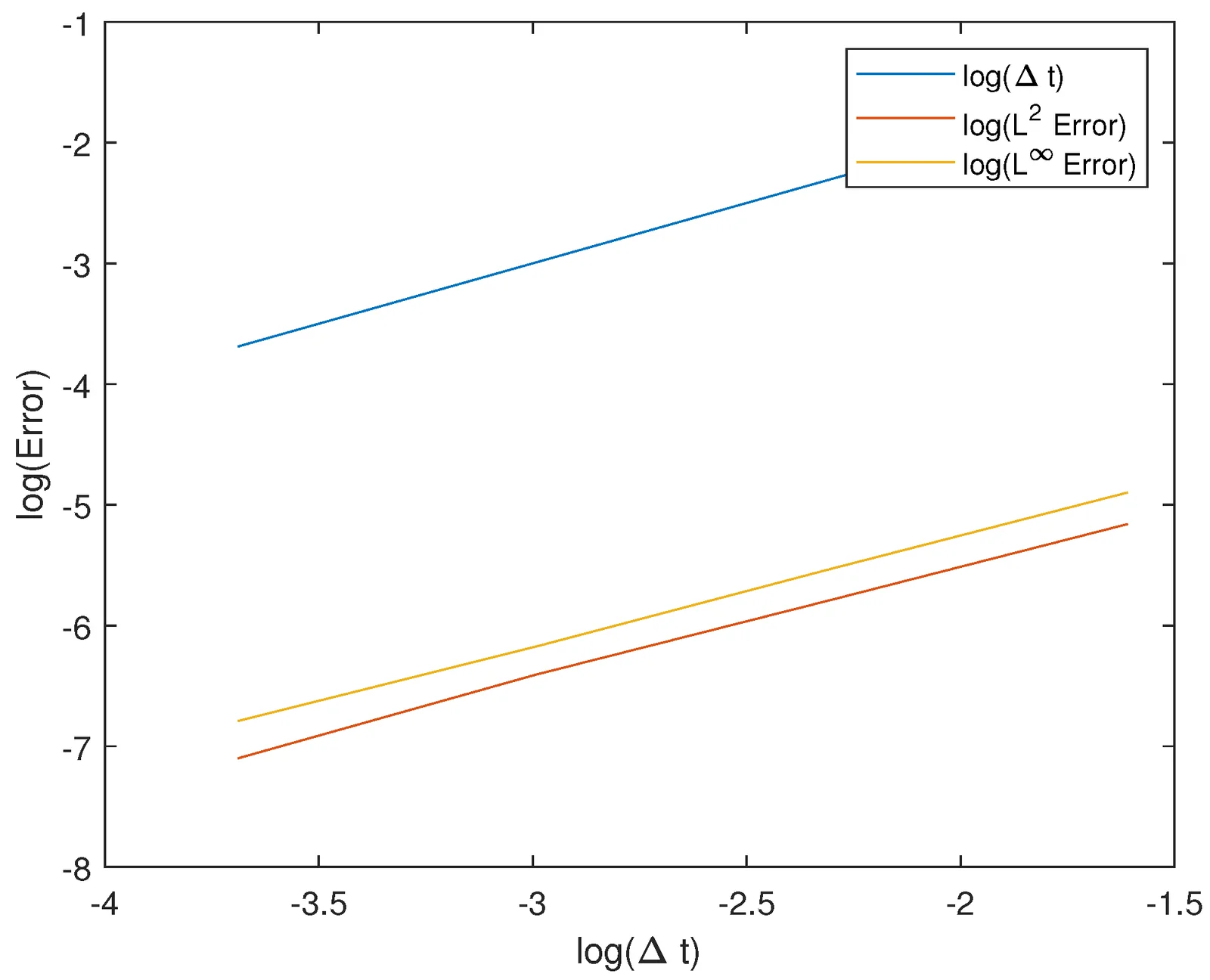
Error in $L^{2}$ and $L^{\infty }$ norm versus Δt, order for α(t)=0.6+0.2cost, SE=40, k=1.

Temporal accuracy is assessed in Figure 1 and Figure 2, corresponding to $\alpha \left(t\right)=\frac{{\left(\sin t\right)}^{2}+2}{5}$ and α(t)=0.6+0.2cost, respectively. A fixed spatial resolution (SE=40) together with piecewise $P^{1}$ elements is employed. The observed temporal convergence rate approaches unity, which agrees well with the theoretical expectation for the time discretization used.

## 5. Conclusions

In this work, we developed and analyzed an interior penalty discontinuous Galerkin (IPDG) method for solving a two-dimensional variable-order fractional mobile–immobile advection–dispersion equation with a divergence-free velocity field. The time discretization was based on a Grünwald–Letnikov approximation combined with a first-order finite difference scheme for the variable-order Caputo derivative, while the spatial discretization employed an IPDG method incorporating an upwind numerical flux for the convective term and an interior penalty formulation for the diffusive part.

Theoretical analysis demonstrated that the proposed fully discrete scheme is unconditionally stable and convergent. Numerical experiments were conducted to assess the accuracy and robustness of the proposed scheme. For sufficiently smooth manufactured solutions, the observed spatial convergence rates are sharper than the conservative theoretical bound and agree with the optimal rates expected for the present interior penalty formulation. In particular, for two distinct velocity fields, V=(1,1) and V=(−x+0.5,y−0.5), using piecewise $P^{1}$ polynomials and several time-dependent fractional orders α(t), the scheme achieves second-order spatial convergence in practice. This behavior is consistent with the smoothness of the test solution and the expected optimal accuracy of the method, although the rigorous theorem provides a more conservative bound. Temporal accuracy tests for $\alpha \left(t\right)=\frac{{\left(\sin t\right)}^{2}+2}{5}$ and α(t)=0.6+0.2cost demonstrated that the convergence rate in time approaches unity, which is consistent with the theoretical prediction.

These results indicate that the proposed IPDG method provides an effective and reliable numerical tool for solving variable-order fractional advection–dispersion problems. Future work may extend the method to higher-dimensional problems, adapt it for non-divergence-free velocity fields, or incorporate adaptive mesh refinement strategies to further improve computational efficiency.

## Figures and Tables

**Table 1:** Spatial accuracy test when $V=\left(1,1\right),\;M=10^{3},\;T=1$ using piecewise $P^{1}$ polynomials.

α(t)	SE	*h*	$L^{2}$-Error	Order	$L^{\infty }$-Error	Order
α(t)=0.8+0.1sint	5	0.304138	6.23284 × 10^−2^	-	1.93859 × 10^−1^	-
	10	0.153613	1.75881 × 10^−2^	1.84	5.26411 × 10^−2^	1.86
	15	0.104167	8.30261 × 10^−3^	1.92	2.50049 × 10^−2^	1.93
	20	0.0760345	4.64527 × 10^−3^	1.93	1.38260 × 10^−2^	1.95
$\alpha \left(t\right)=1-0.5e^{-t}$	5	0.304138	5.82343 × 10^−2^	-	2.10274 × 10^−1^	-
	10	0.153613	1.66073 × 10^−2^	1.81	5.89285 × 10^−2^	1.87
	15	0.104167	6.40942 × 10^−3^	1.96	2.34915 × 10^−2^	1.91
	20	0.0760345	2.86464 × 10^−3^	1.98	1.07726 × 10^−2^	1.97

**Table 2:** Spatial accuracy test when $V=\left(-x+0.5,y-0.5\right),\;M=10^{3},\;T=1$ using piecewise $P^{1}$ polynomials.

α(t)	SE	*h*	$L^{2}$-Error	Order	$L^{\infty }$-Error	Order
α(t)=0.8+0.1sint	5	0.304138	7.23986 × 10^−2^	-	2.21098 × 10^−1^	-
	10	0.153613	1.99731 × 10^−2^	1.79	6.07235 × 10^−2^	1.81
	15	0.104167	9.47678 × 10^−3^	1.88	2.62509 × 10^−2^	1.96
	20	0.0760345	5.08156 × 10^−3^	1.95	1.36693 × 10^−2^	1.97
$\alpha \left(t\right)=1-0.5e^{-t}$	5	0.304138	6.83077 × 10^−2^	-	2.89275 × 10^−1^	-
	10	0.153613	1.89120 × 10^−2^	1.82	7.88518 × 10^−2^	1.83
	15	0.104167	8.73629 × 10^−3^	1.89	3.20161 × 10^−2^	1.95
	20	0.0760345	4.73647 × 10^−3^	1.92	1.61841 × 10^−2^	1.98

## Data Availability

The original contributions presented in this study are included in the article. Further inquiries can be directed to the corresponding author.

## References

[1] Chen Z., Qian J.Z., Zhan H.B., Chen L.W., Luo S.H. (2011). Mobile-immobile model of solute transport through porous and fractured media. Environ. Sci. Eng..

[2] Ding H.F., Li C.P. (2016). High-order compact difference schemes for the modified anomalous sub-diffusion equation. Numer. Methods Partial Differ. Equ..

[3] Sousa E., Li C. (2015). A weighted finite difference method for the fractional diffusion equation based on the Riemann-Liouville derivative. Appl. Numer. Math..

[4] Zhang X., Feng Y., Wei L. (2026). High-Precision Numerical Simulation for Fractional Convection-Diffusion-Reaction Equations. Math. Methods Appl. Sci..

[5] Stynes M., O’Riordan E., Gracia J. (2017). Error analysis of a finite difference method on graded meshes for a time-fractional diffusion equation. SIAM J. Numer. Anal..

[6] Zhang X.D., Feng Y.L., Luo Z.Y., Liu J. (2025). A spatial sixth-order numerical scheme for solving fractional partial differential equation. Appl. Math. Lett..

[7] Zheng X., Wang H. (2021). Optimal-order error estimates of finite element approximations to variable-order time-fractional diffusion equations without regularity assumptions of the true solutions. IMA J. Numer. Anal..

[8] Li C.P., Zeng F.H. (2015). Numerical Methods for Fractional Calculus.

[9] Wang H., Yang D., Zhu S. (2014). Inhomogeneous Dirichlet boundary-value problems of space-fractional diffusion equations and their finite element approximations. SIAM J. Numer. Anal..

[10] Ahmadinia M., Safari Z., Abbasi M. (2020). Local discontinuous Galerkin method for time variable order fractional differential equations with sub-diffusion and super-diffusion. Appl. Numer. Math..

[11] Wei Ŀ., Feng L., Turner I., Mao Z., Liu F. (2026). Numerical investigation of the 2D unsteady natural convection heat transfer equation with tempered fractional constitutive relationship. Commun. Nonlinear Sci. Numer. Simul..

[12] Wei L., Chen Y., Qin F., Huang J., Li P. (2026). The discontinuous Galerkin method for solving the two-dimensional transport equation with distributed-order memory kernel on triangular meshes. Appl. Numer. Math..

[13] Wang N., Wang J., Liu Y., Li H. (2023). Local discontinuous Galerkin method for a nonlocal viscous water wave model. Appl. Numer. Math..

[14] Yang J., Li Y., Liu Z. (2024). A finite difference/Kansa method for the two-dimensional time and space fractional Bloch-Torrey equation. Comput. Math. Appl..

[15] Ju Y., Yang J., Liu Z., Xu Q. (2023). Meshfree methods for the variable-order fractional advection-diffusion equation. Math. Comput. Simul..

[16] Chen S., Shen J., Wang L.L. (2016). Generalized Jacobi functions and their applications to fractional differential equations. Math. Comp..

[17] Li X., Xu C. (2009). A space-time spectral method for the time fractional diffusion equation. SIAM J. Numer. Anal..

[18] Lin Y.M., Xu C.J. (2007). Finite difference/spectral approximations for the time-fractional diffusion equation. J. Comput. Phys..

[19] Liu Y., Zhang M., Li H., Li J. (2017). High-order local discontinuous Galerkin method combined with WSGD-approximation for a fractional sub-diffusion equation. Comput. Math. Appl..

[20] Wang H., Zheng X. (2019). Analysis and numerical solution of a nonlinear variable-order fractional differential equation. Adv. Comput. Math..

[21] Wei L., Yang Y.F. (2021). Optimal order finite difference/local discontinuous Galerkin method for variable-order time-fractional diffusion equation. J. Comput. Appl. Math..

[22] Coimbra C.F.M. (2003). Mechanica with variable-order differential operators. Ann. Phys..

[23] Lorenzo C.F., Hartley T.T. (2009). Variable order and distributed order fractional operators. Nonlinear Dyn..

[24] Ramirez L., Coimbra C. (2010). On the selection and meaning of variable order operators for dynamic modeling. Int. J. Differ. Equ..

[25] Hansen S.K. (2015). Effective ADE models for first-order mobile-immobile solute transport: Limits on validity and modeling implications. Adv. Water Res..

[26] Schumer R., Benson D.A., Meerschaert M.M., Baeumer B. (2003). Fractal mobile/immobile solute transport. Water Resour. Res..

[27] Zhang Y., Benson D.A., Reeves D.M. (2009). Time and space nonlocalities underlying fractional-derivative models: Distinction and literature review of field applications. Adv. Water Resour..

[28] Zhang H., Liu F., Phanikumar M.S., Meerschaert M.M. (2013). A novel numerical method for the time variable fractional order mobile-immobile advection-dispersion model. Comput. Math. Appl..

[29] Jiang W., Liu N. (2017). A numerical method for solving the time variable fractional order mobile-immobile advection-dispersion model. Appl. Numer. Math..

[30] Liu Z.G., Cheng A.J., Li X.L. (2017). A second order finite dfference scheme for quasilinear time fractional parabolic equation based on new fractional derivative. Int. J. Comput. Math..

[31] Golbabai A., Nikan O., Nikazad T. (2019). Numerical Investigation of the Time Fractional Mobile-Immobile Advection-Dispersion Model Arising from Solute Transport in Porous Media. Int. J. Appl. Comput. Math..

[32] Saffarian M., Mohebbi A. (2021). An efficient numerical method for the solution of 2D variable order time fractional mobile-immobile advection-dispersion model. Math. Meth. Appl. Sci..

[33] Sadri K., Aminikhah H. (2021). An efficient numerical method for solving a class of variable-order fractional mobile-immobile advection-dispersion equations and its convergence analysis. Chaos Solitons Fractals.

[34] Podlubny I. (1999). Fractional Differential Equations.

[35] Gao G.H., Sun Z.Z. (2016). Two unconditionally stable and convergent difference schemes with the extrapolation method for the one-dimensional distributed-order differential equations. Numer. Methods Partial Differ. Equ..

[36] Riviere B. (2008). Discontinuous Galerkin Methods for Solving Elliptic and Parabolic Equations: Theory and Implementation.

